# Introduction to a Discussion on the Diagnosis of Tuberculosis

**Published:** 1917-04

**Authors:** Latimer James Short

**Affiliations:** County Tuberculosis Officer for Somerset


*"Scire est nescire, nisi id me*

*Scire alius sciret."*

APRIL, 1917.

### INTRODUCTION TO A DISCUSSION ON THE DIAGNOSIS OF TUBERCULOSIS.

BY

LATIMER JAMES SHORT, M.D., B.S. Lond., M.D. Brist., D.P.H. Cantab.,

*County Tuberculosis Officer for Somerset.*

I PROPOSE to limit my remarks to the early diagnosis of suspected cases of pulmonary tuberculosis in its strictly clinical aspect. This does not mean that I disregard any of the other methods or aids to diagnosis, for if one thing is now certain, it is that every possible aid which can be devised should be used when attempting to make what is frequently, after all, the most difficult diagnosis in medicine. It is extremely difficult, however, for many practitioners, especially those in scattered rural areas, to avail themselves of any but the simplest methods, and that is my reason for concentrating upon those points connected with physical examination which should receive special attention.

Whatever may be our various views upon the numerous methods of treatment now in vogue, it is very significant that every supporter of a particular line of treatment demands for its success that he shall be supplied with the *early* cases, and the importance of getting hold of these is obvious to us all. Unfortunately it is only too common even now to find that a practitioner only notifies a case or attempts to obtain any special treatment for it when it has already passed into stage 3. This appears to be sometimes due to his having treated the case for a long time without even examining the chest at all, or to a lack of appreciation of the significance of anything but the grossest signs. It is true, however, that many patients will not come for medical advice until they have already passed stage 1, and the only remedy for this which has been found to work well in practice is the systematic examination of all contacts living in the house with any notified case. It is usually from these, who may have no apparent symptoms at all, that the early stage 1 cases are found.

Part of the fault lies with medical writers, in that until quite recently the only cases described at all in medical literature are what are really advanced cases, and students are only made acquainted with these gross signs, and consequently when they do not find them in a patient they are inclined to rule tuberculosis out of the case.

It is now accepted that the duration from infection to termination of a case is far longer than was then recognised, and Sir Robert Philip considers that an average of ten years is by no means unlikely. When working systematically upon the school children of Edinburgh, he considered that he could recognise definite signs of tuberculosis by simple physical examination in no less than 30 per cent. of the children. The fact that a large proportion of these very early cases would get well without any treatment is not the question. Admitting this fact, they ought to be recognised and watched.

It may be useful to describe in detail the points which should be noted when dealing with the particular type of case which commonly arouses anxiety on the part of the practitioner, and most frequently leads him to refer the case for a second opinion.

TYPE A.

This group comprises those cases where there are either no symptoms at all, and the case is examined for some other reason, or the signs are the very indefinite ones of general poor health, with either a certain amount of chronic gastric trouble or nervous irritability, with or without a little chronic cough. Here, if there is any sputum at all, it should be carefully tested, if possible by the antiformin method or one of its improvements, and the examination should be repeated several times, recognising that a negative is of no definite value. Also the temperature should be regularly taken and charted, particular notice being directed to the difference between the figure on rising in the morning and at 6.0 or 7.0 p.m. After 7.0 p.m. the temperature is not of so much value.

The chest should be examined in a good light, when the patient has abstained from smoking for some hours, as a certain type of cigarette may cause distinctly confusing signs. On inspection it should be noticed whether the expansion is exactly equal both sides, and whether there is any flatness or hollowing at either apex. On the back a slight thinning of one trapezius should also be noted. On percussion there should be three fingers breadth of resonance above each clavicle, and if any difference, there should normally be slightly more resonance on the left than the right. The resonance also should not be too sharply cut off. Very light percussion is necessary, both above and below the clavicle, and any difference, whether it amounts to dullness or not, must be noted. It is common to get a slight diminution of the note at the base of the same lung, which also suggests a tuberculous lesion.

A very interesting phenomenon recently re-described by Clive Riviere, which is probably purely reflex, and has no connection at all with the actual location of a lesion, is the appearance of two rather narrow horizontal bands of decreased resonance on the back, which he states can always be found if very carefully looked for in cases where there is any definite tuberculous lesion, the diminution being somewhat more marked on the affected side.

On auscultation there should be some alteration of the breath-sounds, either harshness or diminution, exactly corresponding to the site of the altered percussion note previously discovered, and any adventitious sounds, whatever their nature, must also correspond to the position of the dullness and altered breath-sounds. Sounds which do not correspond to other signs must not be given serious diagnostic value, but when they do correspond, even if they are exceedingly slight, they are of very serious import.

A very early sign insisted on by Sir Robert Philip is the presence of a chain of very small glands running down the anterior border of the sterno-mastoid. Posterior glands are of no value. A slight degree of myotatic irritability on the suspected side is a usual confirmatory sign.

### TYPE B.

*Chronic Bronchitis or Asthma, with some loss of flesh.*—In this case the sputum must be *repeatedly* examined, as it is often the most important factor. Also, if the case is really one of tuberculosis, there must be some localised alteration in the percussion note, which is not given with a case of non-tuberculous bronchitis.

### TYPE C.

*Acute Pneumonia or Influenza.*—Frequently at first one can do nothing but watch the case carefully and take regular four-hourly temperature charts. If tuberculous, there will very soon be apparent a definite swing of at least $1\frac{1}{2}$ to 2 degrees. It will also be found that the respiration rate is not increased correspondingly with the temperature, and that the patient's mental condition is far too clear or unconcerned. In fact, the more cheerful the patient with a high temperature the more likely is it to be tuberculosis. Within a week it is nearly always possible to find a definite localised alteration of the percussion note if the case is tuberculous.

### TYPE D.

*Typhoid or mental symptoms.*—Here the suggestion is that the case is one of general or miliary tuberculosis, and the temperature may be either very irregular or sub-normal. Typhoid should always, of course, be thought of and watched for, but if general tuberculosis is present, the patient's mental condition is usually much duller than the temperature would suggest. It is possible to find some slight alteration in the percussion note in the chest, and usually the " feel " of the abdomen is quite distinctive.

### TYPE E.

*Pleurisy.*—Where there is any clear fluid in the chest, and the cause is not quite obvious, it is tuberculous in 90 per cent. of cases, and the patient should be treated as such straight away, even if there are no signs of any other tuberculous lesion.

### TYPE F.

Owing to the war a new difficulty has arisen, that in cases of gas poisoning, seen some weeks after the event, may present signs very strongly resembling pulmonary tuberculosis. It is not quite certain what causes the signs, but there can be very definite areas of localised dullness, with harsh breathing and even crepitations to match. Even if these are at the apex, however, it is usually possible to find some areas in other parts of the lung, and as the patient has no temperature and the sputum is always negative, a diagnosis can eventually be made. Such cases, however, should be closely watched before a definite opinion is given, as it has happened that gas poisoning has lighted up an old infection which was previously unknown to the patient.

Experience has led me to the opinion that the above-described types of cases are those which cause most anxiety to practitioners, and I have tried to indicate the points on which I lay special value when such a case is referred to me for an opinion.

---

J. M. H. MUNRO, D.Sc. Lond., LIEUT. AND M.O. SOM. VOL. REGT.

---

I have listened to Dr. Short's opening speech with the greatest pleasure and profit. A portion of the subject-matter was new to me, and a portion traversed familiar ground. For the former I am grateful, and with the latter I cannot find a single item of disagreement. For this reason, and because Dr. Short has treated so fully of the clinical examination, I shall be expected to deal rather with the laboratory aids to diagnosis. I carry about with me a somewhat heretical though strong impression that in the diagnosis of tuberculosis the consultant who devotes the greater part of an hour to inspection, palpation, auscultation, and percussion is at a great disadvantage compared with the general practitioner who can watch the patient under varying conditions.

Reflect for a moment on the difficulty often experienced in undoubted pulmonary cases in determining whether the lesion is absolutely limited to one lung. Here we are forced to rely on the usually superior clinical skill of the consultant, and neither laboratory methods nor the more ample opportunities of the practitioner in charge count.

Great as my hesitation often is when asked for an opinion after one examination whether such and such a young person is tubercular, I never feel otherwise than confident if I am allowed to watch the case for a time, and one rarely has to employ all the available methods before coming to a decision.

Of the ordinary aids to diagnosis the temperature chart, valuable as everyone admits it to be, is susceptible of further refinement. A naval officer, whose case I have already described,[1] invalided twice for spells of pyrexia, which subsided sufficiently under two or three weeks' rest in hospital. He came to me complaining of tiredness on exertion, quite a novelty for a man of his strength and activity. There was little else to go on. The most careful and repeated chest examinations left one in doubt ; an excellent skiagraph received opposite interpretations from two experts ; there was no sputum, and the officer had to return to duty. He kept a morning and evening temperature chart while on service, and on next leave again consulted me. On the strength of this chart and a suspicion as to one apex recorded by one of the several naval surgeons who had examined him, he obtained extended sick leave, and placed himself in my hands. Although the chest signs were still slight or wanting, the temperature was sensitive to exertion. An almost complete rest was ordered at first. For twelve months, having little else to do, he kept a most accurate and painstaking two-hourly (when awake) temperature chart, which, although limited in range, was characteristic. Very minute therapeutic doses of tuberculin (T.R.) were given at considerable intervals ; they were such as would make no impression on an ordinary morning and evening temperature chart, but on the two-hourly one the slight temporary rise due to each was clearly shown.

[1] "A Disputed Case of Phthisis," read before the Bath Clinical Society, 1914.

After a time graduated exercise, still regulated by thermometer, was ordered, and the chest became almost normal. After another extension of leave, the case, which in its course was reviewed by no less than twelve Naval and Fleet Surgeons, came up for the important final decision, whether to invalid out of the Service for tuberculosis or to repudiate the tubercular diagnosis. The unique temperature chart, I believe, was largely responsible for the decision that the patient had been suffering from pulmonary tuberculosis, but was now cured. He was retained in the Service, and has endured all the exposure of patrol work in this war without suffering. The daily range of temperature is more important than the mean line. To emphasise this, and at the same time save space, I some years ago charted my tubercular patients in parallel vertical lines only, one for each day, the length denoting the extreme range. The chart thus forms a ribbon of varying width. Great and varying width, especially in regular waves, is as characteristic of tuberculosis as any one sign can be.

But it must not be forgotten that in rare cases the wide fluctuation is almost entirely below 98.4, the temperature rarely rising to 99. A retired doctor of great experience and quite unusual skill in examination brought me his married daughter with a chart of this description, which he said negatived the idea of tuberculosis, as there was no pyrexia, and she was habitually subnormal. She had been ailing a little, and he demonstrated to me slight right apical signs which might easily have escaped another observer. A skiagraph was rather reassuring as to the lungs, but showed a bronchial gland. Under treatment and a summer in Scotland health was re-established, and the temperature, which he had regarded as idiosyncratically subnormal, became perfectly normal.

Coming to laboratory methods of diagnosis, I am convinced that in the future it is to these we shall have recourse if we are to attain any great success in combating tuberculosis at its inception.

The oldest laboratory test of all in this connection—examination of sputum for T.B.—has already rendered incalculable service by detecting tuberculosis in a fairly early stage (from a clinical standpoint), and in the cases following whooping-cough, bronchitis, and other catarrhal affections. In distinguishing from tuberculosis the broncho-pneumonic conditions giving rise to dubious clinical signs, we have in addition to the absence of T.B. from the sputum confirmation by the presence of the pneumococcus and the micrococcus catarrhalis; many casesof bronchitis, broncho-pneumonia, and "influenza" being simply acute or chronic infections of a portion of the respiratory tract by these two organisms.

The distinction is very important, because on it hinges divergence of treatment; and the diagnosis is difficult, especially in cases where (as I found with a relative of my own on my recent return from France) a relapsing pneumococcal catarrh re-awakens crepitations over the site of a healed tubercular cavity. But this method carries us no farther than the recognition or confirmation of a comparatively advanced stage of a tubercular lesion, for I regard as advanced the tiniest patch which the most expert clinician can demonstrate by auscultation and percussion. And the emphasis with which Dr. Short rightly enforces the urgency of an early *clinical* recognition of tuberculosis should, I think, attach to an equally urgent demand for a laboratory diagnostic sensitive to a *really* early stage of infection, one that would give us, say, 95 per cent. of cures with existing modes of treatment. In what direction may we look for this?

Of the rough-and-ready methods based on the tubercular re-action—Calmette's Conjunctival Test, Von Pirquet's and

Moro's Skin Tests—I can only say that I very rarely employ them, and would advise those who think of doing so to read paper by my colleague, Dr. Leckie, in the *British Medical Journal May* 16*th*, 1914

When I wished, some two years before the war, to subject to parallel tests for tubercular and syphilitic complication or causation the numerous multi-arthritic cases admitted to the Royal Mineral Water Hospital (mostly shovelled in under the name of rheumatoid), I set these tests aside in favour of the temperature reaction to Koch's old tuberculin. This has the sanction of universal use in veterinary practice, and the slaughter of animals reacting to it has provided in the autopsies ample proof of its reliability. Testing in this way 70 cases with successive doses at a few days' interval of 1/1000th, 1/500th, 1/250th c.c. of O.T.—I never use larger ones—no less than 36 cases reacted with a rise of 1 °F. or more within forty-eight hours. If of these we consider only the 21 cases which reacted to the first dose, it appears to me incontrovertible that they contained active tubercular foci, either in the affected joints or somewhere else in the body, and that in either case valuable indications for treatment were obtained. All the cases had undergone careful clinical examination, and neither from this nor from their histories would tuberculosis have been inferred, excepting from one history of a scraped tarsus, and perhaps from one effusion into an olecranon bursa (this was absorbed after the first diagnostic dose of tuberculin). In suspected pulmonary phthisis I think great caution should be used in selecting cases to be tested in this way, and those in which I have personally used it might be counted on the fingers of one hand. There are two objections which seem valid: the dose given as the minimum for diagnostic results may yet be too large from a therapeutic point of view, and may set up an unfavourable turn; and, secondly, it may induce a hypersensitiveness to the much smaller doses of tuberculin which one may contemplate as a course of treatment.

The war, which summarily put a stop to the investigations I have alluded to, has shown how laboratory methods of diagnosis may be exalted and refined to cope with an urgent necessity.

Anti-typhoid inoculation was general in 1914 in our Army, and as late as January, 1916, I was told of a large War Hospital in a distant county where the two pathologists, playing cards to pass the time, explained to my informant that there was little to do except a few sputa, because " the Widal test, you know, is of no use with these inoculated chaps."

Meantime, under the stress of necessity, what had happened at the front ? Typhosus inoculation had been found effective as against typhoid, but the enterics coming into infectious diseases hospitals comprised a large percentage of Paratyphoid B. cases, and a considerable percentage of Paratyphoid A. As a consequence, inoculation with a triple vaccine, T.A.B., superseded the single inoculation in the British Army in the autumn of 1915, the French in January, 1916, and the Belgian in October, 1916. The bacteriology of the blood, stools, and urine as mainly responsible for these advances ; but a less laborious and more uniformly successful means of diagnosis was urgently wanted, and was soon supplied by Professor Dreyer's brilliant work. He made the quantitative technique of the Widal test so simple and certain that reliable day by day curves of the agglutinating power of the patient's serum to standard cultures of typhosus, Para. A. and Para. B., could be obtained, in which the effects of inoculation, infection, and stimulation could all be traced, and in the majority of cases the diagnosis could be made from the curves alone. When I took up work at the Anglo-Belgian Hospital receiving all the infectious cases from the Belgian front, I found that with typhosus-inoculated patients the method had for months almost superseded blood cultures and platings as a means of diagnosis. During my stay the T.A.B. inoculation was made compulsory, and I had the interesting experience of dealing with forty to fifty patients who developed enteric within a few days or weeks of the triple inoculation. Many of these had been infected just before inoculation, and the rest before the development of protection. Now these are the most puzzling of all to diagnose by serum examination, because the three inoculation curves are in full career, to these are superadded the true infection curve, and on this again is superimposed the group stimulation curve. I brought back with me all the charts of these special cases, which I had the pleasure of submitting to Dreyer, and in some test cases the diagnoses agreed to by us were confirmed by the results of plating the stools.[1]

The moral of this digression is that if blood-testing can be made to unravel so complicated a problem as that of the enteric group of infections, it is probably to it we must also look for a reliable and *early* (in the bacteriological sense) diagnosis of a tubercular infection. And were the urgency of the need as great as that caused by a war emergency, it is safe to say the problem would soon be solved. But as, apart from war needs, bacteriological research is about the last object in the world on which individuals or hospitals (not to

[1] The average number of enterics in the hospital rarely exceeded 100, but to place the daily agglutination testings on a basis of clockwork regularity an organised laboratory staff was necessary. This consisted of myself, a thoroughly competent and zealous chief assistant, a second assistant trained by him to be equally expert at this particular work, two orderlies at stated hours to do all the washing-up, a medical officer (generally the O.C.) to save my time by doing the vein-punctures for the day, and the dispenser to clean, sterilise, and supply the needles. The results were calculated and tabulated by the registrar.

speak of County Councils) will spend a superfluous halfpenny, I am not sanguine as to the immediate future. The agglutination reaction has certain difficulties in its application to the tubercle bacillus. The complement-fixation test promises much more, and if carried out, say, with the scrupulous technique described in Besson's work on bacteriological method will often be successful; but we need the volumes of work to be done on it in its application to tubercle that have been lavished on its application to the diagnosis of syphilis before we shall really know "where we are." With it remains the opsonic index, and in spite of neglect and a certain amount of obloquy, I am by no means certain it is not even now the best means we have of making an early diagnosis. As a guide to dosage in tuberculin treatment I have never employed or advocated it on an extensive scale, for reasons into which I need not enter. But I have had so many successes with it as a diagnostic method, that I always fall back on it when baffled in simpler indications. The technique requires a much greater amount of skill than Dreyer's agglutination method for enterics, but on the other hand yields results of greater quantitative accuracy. The main claims originally made, namely that the index is uniform and almost stationary in uninfected persons, variable from day to day in many infections, sensitive to tuberculin, to exercise, to massage, in all infections, are quite uncontroverted, and are verifiable by anyone who chooses to take the trouble to acquire the necessary skill. And I venture to say that in my opinion if all young people of suspicious family antecedents found to have a variable opsonic index to the tubercle bacillus were to be inoculated therapeutically with *small* doses of suitable tuberculin at intervals of a few weeks, more would be done in the way of cutting short really incipient tuberculosis than we can hope to accomplish by treatment of any sort directed to the advanced lesions revealed by auscultation and percussion.[1]

---

### J. C. MacWatters, M.R.C.S., L.R.C.P.

---

J. Courtenay MacWatters stated that in his practice he had learned to place very little reliance on the specific local reactions of Calmette, Von Pirquet, and Moro. The first was, he believed, attended with very serious risks, and the others were unreliable, chiefly on account of the fact that they were too sensitive, that such a large number of persons "reacted" who were free from active tuberculous disease, the explanation being that at some date they had been invaded by the Tubercle Bacillus and had developed the specific antibodies.

He expressed it as his practice to depend on the careful reviewing of all the points in the history of a suspected case, in conjunction with the most careful and extended observance of physical signs.

He thought the presence of infraclavicular myotatic irritability was a sign of very great value when more marked on one side.

As regards any specific reaction methods in diagnosis, he was quite convinced that the Opsonic Index was of the greatest value when a sequence of at least three observations were obtained before and after eliciting a specific response, either by inoculation with Tuberculin "B.E.," or by active disturbance of the circulation of the tissues suspected by exercise or massage, etc., any variation above 1.2 or below

[1] I would like to see arranged tests of the sera of a hundred such young people, without seeing them, *pari passu* with clinical examinations by an expert like Dr. Short. Though not a sporting man, I am open to wager (1) that a greater number of positives would be returned by me than by him, (2) that substantially all his positives would be returned positive by me.

0.8 being taken as very strong evidence of active tuberculous processes being present.

In early phthisis of children, where it was difficult to obtain sputum for examination, he had frequently been able to obtain the desired material by holding a small piece of gauze in a pair of forceps, or on the end of a wire, in the back of the throat while the child was induced to cough. By this means it was as a rule possible to obtain a specimen of phlegm adhering to the gauze from even a very young child.

He drew attention to those cases of chronic pneumococcal infection of the chest where the physical signs were strongly suggestive of phthisis, and laid some stress on the general appearance of the majority of phthisic children, their complexion, skin, eyelashes, and sclerotics, and on the presence of enlarged supraclavicular lymphatic glands most marked on one side even before definite signs were discoverable in the chest.

He concurred with Dr. Munro's opinion that as a rule where phthisis was discoverable by physical signs the disease was probably well in progress, and that it was only by specific blood examinations that we could hope to deal with the truly initial stages of tubercular infections.

---

H. Carleton, M.D., Capt. R.A.M.C.

---

1. The conception of early tuberculosis as a purely apical disease is probably untrue. The idea rests largely on the basis of *post-mortem* findings, which are not records of early pulmonary tuberculosis.

2. Appearances of good radiographs strongly support the view that early infection begins at and spreads centrifugally from the hilum of the lung, either as a retrograde spread through the peribronchial lymphatics or as a perbronchial aspiration infection.

3. This view of spread from the hilum is supported by consideration of the pathology of primary tuberculous pleurisy. The subpleural plexus of lymphatics connect for the most part directly with the lymphatics at the root of the lungs, and have but little connection with the peri-and inter-lobular lymphatics. Such pleurisies find their origin in disease at the root of the lung.

4. Spread against the lymph stream is no serious difficulty in accepting the above view, and it accounts for the gradual onset of the disease in most cases.

5. Of bacteriological methods, useful in early diagnosis, the specific precipitin reaction deserves further notice. As employed by Karl Spengler it is probably beset with fallacies.

